# The Uses of the Tongue

**Published:** 1874-02

**Authors:** 


HALL'S

### JOURNAL OF HEALTH

AND

MISCELLANY.

VOL. XXI.—FEBRUARY, 1874.—No. II.

### THE USES OF THE TONGUE.

To TASTE and talk, of course! It does neither. An ulcerated tongue was lately cut entirely out at the Royal Free Hospital in London. Within a week the man was heard distinctly to say, "I should like some more beef tea." Blindfold a man, ask him to open his mouth wide and keep it open, put salt on his tongue, or a drop of wormwood oil; he cannot tell the difference.

Take half a teacupful of ice water, pour it into the centre of a dose of castor oil; open the mouth, put the rim of the cup far back on the tongue, toss up the cup, down goes the oil without a taste of it, as long as you keep your mouth open, or do not allow the oil to touch the lips. Is taste then in the lips?

But reader, where have you been all your life that you should know no better than to believe that the sense of taste is in the tongue or the lips? This may show how unobservant most persons are. The great and lamented Agassiz was brought by a painter to look at a fish; the artist himself thought it was as near perfection as possible. In an instant the philosopher said to him, "Don't you see you have left out three scales." To produce the sensation of taste, the tongue, the lips and the substance must all come in contact at the same time, or in the process of smacking the lips.

